# Supplementary material for: In vivo quantitative imaging of tumor pH by nanosonophore assisted multispectral photoacoustic imaging
Source: Nat Commun. 2017 Sep 7;8:471. doi: 10.1038/s41467-017-00598-1 (PMC5589864; doi:10.1038/s41467-017-00598-1)
Supplement: Supplementary file 1 — Supplementary Information [file 41467_2017_598_MOESM1_ESM.pdf]

Description of Supplementary Files

File name: Supplementary Information
Description: Supplementary figures, supplementary methods and supplementary references.

File name: Peer review file

Supplementary Methods

Characterization of SNARF-PAA NP

The blank PAA NP solutions are optically clear in the visible spectrum, as shown in the photograph in **Supplementary Fig. 1a** in comparison with the PAA NPs containing SNARF-5F. As checked by the UV-VIS spectrometer, the optical spectrum of blank PAA NP solution is significantly weaker than that of SNARF-PAA NPs in the wavelength range of 565-600 nm, as shown in **Supplementary Fig. 1b**. This weak spectrum of blank PAA NPs measured by the UV-VIS, instead of reflecting their optical absorption, should mostly come from their optical scattering of the lower wavelength light.

A Philips CM-100 transmission electron microscope (TEM) was used for dried size determination by negative staining using uranyl acetate. A Dynamic Light Scattering instrument (DLS, Delsa Nano C particle analyzer instrument, Beckman Coulter) was used to determine the hydrodynamic particle size and zeta potential. The hydrodynamic size of surface modified SNARF-PAA NP was ~60 nm (optimum for passive targeting towards tumor); while the dehydrated NP size was ~17 nm (**Supplementary Figs. 2a** and **2b**). The sizes of naked SNARF-PAA NPs did not differ significantly from surface modified SNARF-PAA NPs. Zeta potential (surface charge) dropped from 26±2.3 mV to 17±1.2 mV as a result of surface modification. The zeta potential was slightly reduced because of the PEGylated surface.

To test the cytotoxicity, 9L rat glioma cell line (American Type Culture Collection) was cultured in RPMI 1640 medium supplemented with 10% Fetal Bovine Serum (FBS) and 1% Antibiotic-antimycotic. Approximately 10,000 cells were seeded on 96 well plates. SNARF-5F, blank PAA NPs, and SNARF-PAA NPs were dissolved in the culture medium (20 mg ml^{-1}). The concentration of free SNARF-5F solution was equivalent to that loaded in SNARF-PAA NPs. Appropriate amounts of SNARF-5F, blank PAA NPs, or SNARF-PAA NPs were added and diluted with fresh culture medium to make final concentrations of 0.2 mg ml^{-1}, 1 mg ml^{-1}, and 5 mg ml^{-1} in each well. Wells that contained cells without being treated with SNARF-5F or SNARF-PAA NPs served as the control. After overnight incubation, the culture medium in each well were carefully removed and replaced with 100 μL of colourless RPMI. Then, 20 μL of (3-(4,5-Dimethylthiazol-2-yl)-2,5-Diphenyltetrazolium Bromide) (MTT) (5 mg ml^{-1}) was added into each well and incubated at 37°C for 4 hours. In order to dissolve insoluble MTT formazan product, 100 μL of DMSO was added into each well and placed at room temperature for 2 hours. The cell viability was analyzed, by measuring absorbance at 590 nm (reference 620 nm), by an Anthos 2010 Microplate Absorbance Reader (Biochrom Ltd.) (n=3). The MTT assay result (**Supplementary Fig. 2c**) demonstrates that none of free SNARF-5F, blank PAA NPs, or SNARF-PAA NPs exhibits any significant cytotoxicity at the concentrations tested.

Based on our former studies, the response time of our PAA NP based nanosensors can vary, depending on the nanoparticle composition and matrix[1]. To study the kinetic response of our SNARF-PAA NPs to the change of environmental pH level, we have conducted a time-dependent fluorescence measurement taken by Horiba Fluoromax-3. The fluorescence measurement of a SNARF-PAA NP solution (540 nm ex/580 nm em) was continuously taken (once every second) over a period of 1000 seconds. During the measurement, the solution was

kept stirred using magnetic stirrer equipped within the fluorometer. To change the pH level in the solution, small volumes of 1M HCl or 1M NaOH were added alternatively (setting the solution at pH 6, pH 7 or pH 8). As shown in **Supplementary Fig. 3**, the fluorescence measurement as a function of time demonstrated fast optical response of the SNARF-PAA NPs to the pH change in the solution medium. The fluorescent signal from the SNARF-PAA NPs responded within 1 second after adding of either HCl or NaOH, and stabilized in a few seconds. The time needed for signal stabilization is due to the limited stirring speed. This result, besides validating the quick kinetics of the SNARF-PAA NPs, also confirmed their reversibility in sensing the pH level.

To study the photostability, a SNARF-PAA NP solution (2mg ml^{-1}, in PBS pH 7.4) was continuously illuminated with a laser beam at 565-nm wavelength and 10 mJ cm^{-2} light fluence over a total time period of 60 min. The optical absorption of the solution at different time points (i.e. 15 min, 30 min, and 60 min) was measured by the UV-VIS spectrometer. The relative percentage change in optical absorption was used to study the photobleaching. Although it has been known that SNARF-5F can be photobleached, the photostability of the SNARF-PAA NPs, as demonstrated by **Supplementary Fig. 4**, is satisfactory. At 15 min after illumination (accumulated light dose 90 J cm^{-2}), the change in optical absorption is about 2% which further increased to about 10% at 60 min (accumulated light dose 360 J cm^{-2}). This slow speed of photobleaching is highly desirable for potential *in vivo* applications. Using our current system, quad-wavelength ratiometric PAI of a tumor takes less than 1 min for image acquisition at all the four wavelengths. The estimated photobleaching during this time period is less than 0.2%.

To study the potential change of the optical absorption spectrum of SNARF-PAA NPs with temperature, we have generated calibration curves at different temperatures, including 25°C (room temperature), 37°C (body temperature), 43°C, and 48°C. Each calibration curve reflects the ratio between the absorbance at 600 nm and 565 nm (i.e. A_{600nm}/A_{565nm}) as a function of the pH level. As shown in **Supplementary Fig. 5**, there is no obvious difference among the calibration curves at different temperatures, demonstrating good temperature stability of SNARF-PAA NPs.

Optical absorption spectra of free SNARF-5F dye

The optical absorption spectrum of free SNARF-5F dye as a function of pH level is shown in **Supplementary Fig. 6a**. Similar to its emission spectrum which undergoes a pH-dependent wavelength shift, the absorption spectrum of SNARF-5F also changes with pH. When human serum albumin was added in the SNARF-5F solution, the interaction between SNARF-5F dye and proteins can change the optical absorption spectra of the dye, as shown in **Supplementary Fig. 6b**.

SNARF-PAA NP accumulations in organs and tumors

The SNARF-PAA NP's accumulations in organs were studied on a mouse model. As soon as the last sets of images were taken, the mice were sacrificed. Then, the organs and the tumor tissues were isolated and kept in 10% buffered formalin acetate solution for 24 hrs. The tissues were dissected into thin slices and the fluorescence images (ex 560 nm/em 630 nm) were taken using Leica DMIRB inverted fluorescence microscope. The fluorescence intensities

were quantified using MATLAB. As shown in **Supplementary Fig. 7**, SNARF-PAA NPs were mostly accumulated in the tumors and the livers. The liver accumulation is common for intravenously injected nanoparticles that are 50-100 nm in size.

Error in pH quantification due to optical attenuation

We estimated the potential error in pH quantification due to the optical attenuation at different wavelengths for quad-wavelength PA ratiometric imaging. A line target (a black mental wire) was covered by slices of chicken breast tissue with different thicknesses (depths=2 mm, 4 mm, 6mm, and 10 mm, respectively), and measured at the four wavelengths (i.e. 565 nm, 576 nm, 584 nm, and 600 nm). The PA signal amplitude at each wavelength was then normalized respectively by the signal amplitude acquired from the target without being covered (i.e. depth=0), as shown in **Supplementary Fig. 8a**. At 2 mm or 4 mm depth, the difference among the normalized PA signal amplitudes at the four wavelengths were small (<1%). At 6 mm depth which was the maximum tumor depth for the animal model involved in this study, the normalized PA signal amplitudes at 600 nm and 565 nm showed a difference of 4.8% as a result of the wavelength-dependent optical attenuation. Without compensating this 4.8% difference, the estimated error in pH quantification based on ratiometric PAI is 0.16 pH. When tissue depth was further increased to 10 mm, a larger difference of 10% was observed between the normalized PA signal amplitudes at 600 nm and 565 nm. Without compensating this 10% difference, the estimated error in PA pH imaging is 0.34 pH. **Supplementary Fig. 8b** shows the estimated error in pH measurement as a function of the imaging depth. As expected, the error becomes larger when the imaging depth increases. This error in pH quantification due to the spectroscopic difference in optical attenuation in tissue can potentially be compensated by considering the optical fluence in the simulation for quad-wavelength ratiometric PAI.

Sensitivity of PAI of SNARF-PAA NPs

An experiment has been conducted to assess the sensitivity limit of PA imaging in detecting the SNARF-PAA NPs in subsurface tissue. The SNARF-PAA NP solutions (in PBS pH 7.4) were placed in an optically transparent tube (1.8 mm inner diameter) which was covered by a slab of 6-mm thick chicken breast tissue. The surface of the chicken breast was illuminated with a homogenous light beam with light fluence of 20 mJ cm^{-2} at 565-nm wavelength. The SNARF-PAA NP solutions were diluted to different concentrations, including 0.05 mg ml^{-1}, 0.1 mg ml^{-1}, and 0.2 mg ml^{-1}. The PA signals detected from the NP solutions are shown in **Supplementary Fig. 9**. At even very low concentration of 0.05 mg ml^{-1} which is 400 times dilution of the injection concentration, the NP solution was still detectable with a good signal-to-noise ratio over 20 dB. 400 times of dilution was decided because we estimated that the tumor volume was around 1/400 of the mouse body and assumed SNARF-PAA NPs were uniformly distributed in the body. Considering the preferential accumulation of the NPs in the tumor due to both enhanced permeability and retention (EPR) effect and the active tumor targeting moiety, the actually NP concentrations in the mouse tumors *in vivo* should be higher.

Quad-wavelength ratiometric calibration of the PAI system

Gel phantoms (1.6-mm cylinders) contained SNARF-PAA NPs (concentration 10 mg ml^{-1}) at different pH buffers (i.e. pH 6.2, pH 6.6, pH 7, and pH 7.4) were made. Each phantom was imaged along the cross section using the PAI system built on the Verasonics US platform at the four wavelengths (565 nm, 576 nm, 584 nm, and 600 nm). For the PA image at each wavelength, the pixel intensities in the phantom were averaged. The average PA image intensities at the three wavelengths (576 nm, 584 nm and 600 nm) were divided by the average PA image intensity at the isosbestic point (565 nm). For each ratiometric measurement at each pH level, the measurements from 50 images were averages and the standard deviation was calculated, as shown in **Supplementary Fig. 10**. Linear fittings of the measurements at the four pH levels were conducted, and the resulted calibration lines (i.e. 576 nm/565 nm, 584 nm/565 nm, and 600 nm/565 nm) were compared to those from the V312 transducer. The calibration lines from the two systems are similar.

Hemoglobin oxygen saturation (sO_2) images before the injection of SNARF-PAA NPs

The quad-wavelength PA ratiometric imaging provided hemodynamic functional imaging including both hemoglobin oxygen saturation (sO_2) and total hemoglobin concentration (THb) at the same time of pH imaging, as shown in **Figs. 5c** and **5d**. We also studied the sO_2 image of the same tumor at 0 min (i.e. before the injection of SNARF-PAA NPs), as shown in **Supplementary Fig. 11**. This image at 0 min shows similar sO_2 level as the image acquired at 75 min. The center area of the tumor also has relatively low oxygen saturation.

Supplementary Figures

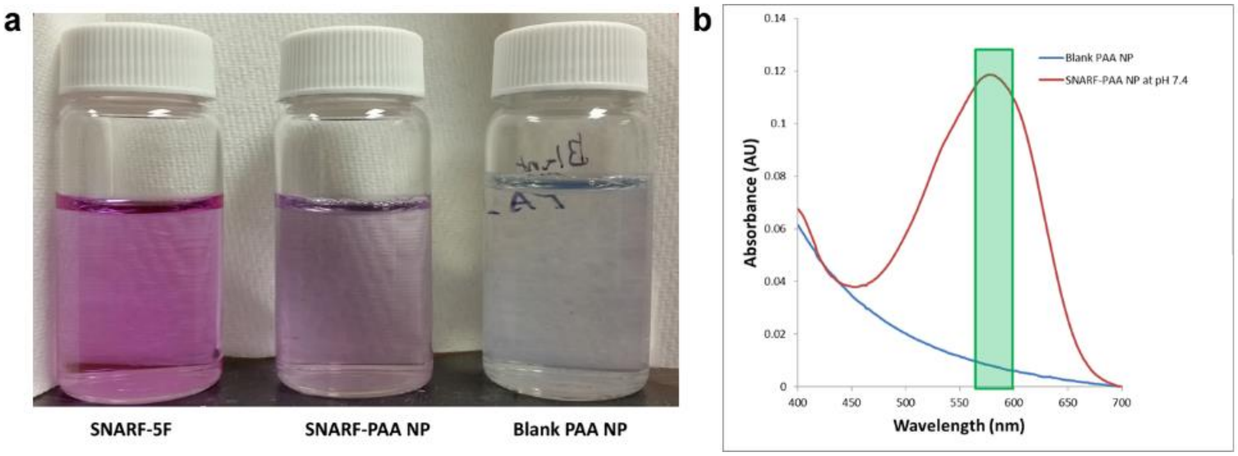

Supplementary Figure 1: (a) Photo showing SNARF-5F, SNARF-PAA NP, and blank PAA NP solution (2mg ml^{-1} or equivalent dye concentration in PBS pH 7.4). (b) UV-VIS spectra of blank PAA NPs and SNARF-PAA NPs at equal concentration (2 mg ml^{-1}). The green rectangle indicates the range of the optical wavelengths (i.e. 565-600 nm) for quad-wavelength ratiometric PAI of tumor pH.

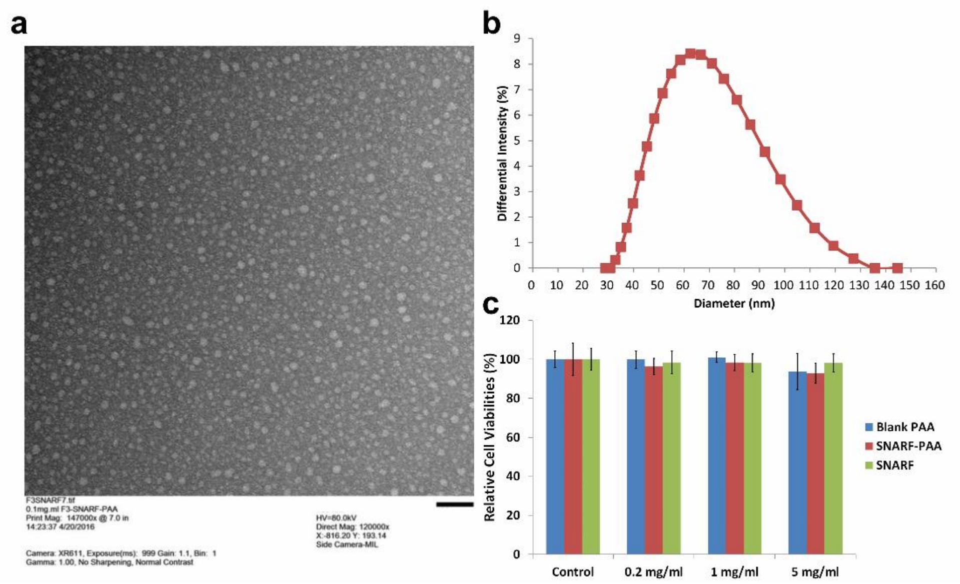

Supplementary Figure 2: SNARF-PAA NP size and toxicity characterizations. (a) Transmission Electron Microscopy image of SNARF-PAA NPs. Scale bar, 100 nm. (b) Hydrodynamic size measurements of SNARF-PAA NPs using Dynamic Light Scattering. (c) *In vitro* cytotoxicity test of free SNARF-5F dye, blank PAA NPs, and SNARF-PAA NPs. The concentration marked was based on SNARF-PAA NP concentration. The concentration of SNARF-5F dye is equivalent to that loaded in SNARF-PAA NPs. Error bars represent standard deviations (*n*=3).

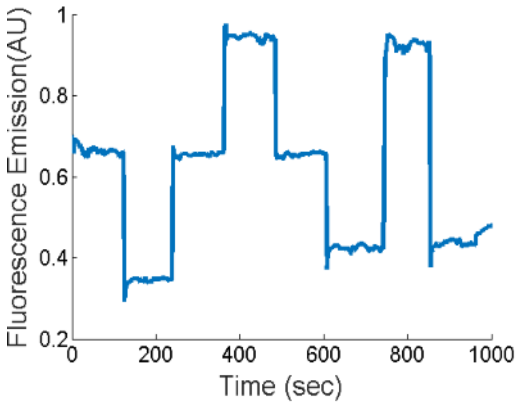

Supplementary Figure 3: Time-dependent fluorescence measurement (540 nm ex/580 nm em) demonstrating the kinetics of SNARF-PAA NPs in sensing the environmental pH change.

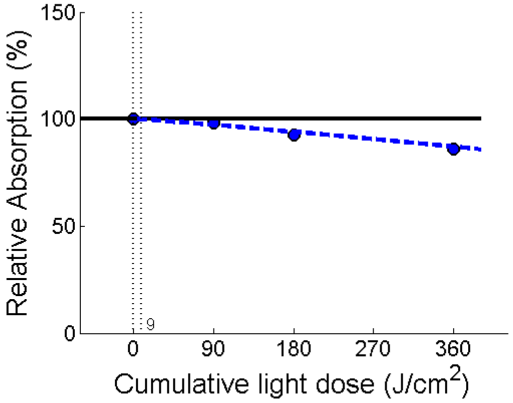

Supplementary Figure 4: Photostability of SNARF-PAA NPs. The optical absorption of SNARF-PAA NP solution (2mg ml^{-1} in PBS pH 7.4) was measured at different time points during a total period of 60 min when the solution was continuously illuminated with 565 nm laser beam with homogeneous light fluence of 10 mJ cm^{-2}. The relative absorption of the solution is presented as a function of the cumulative light dose.

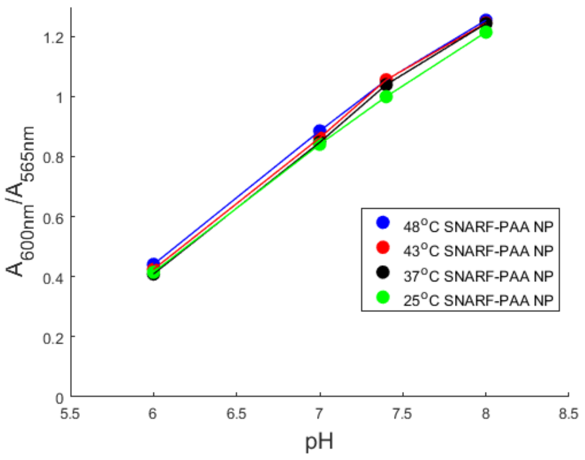

Supplementary Figure 5: Calibration curves of SNARF-PAA NPs at different temperatures.

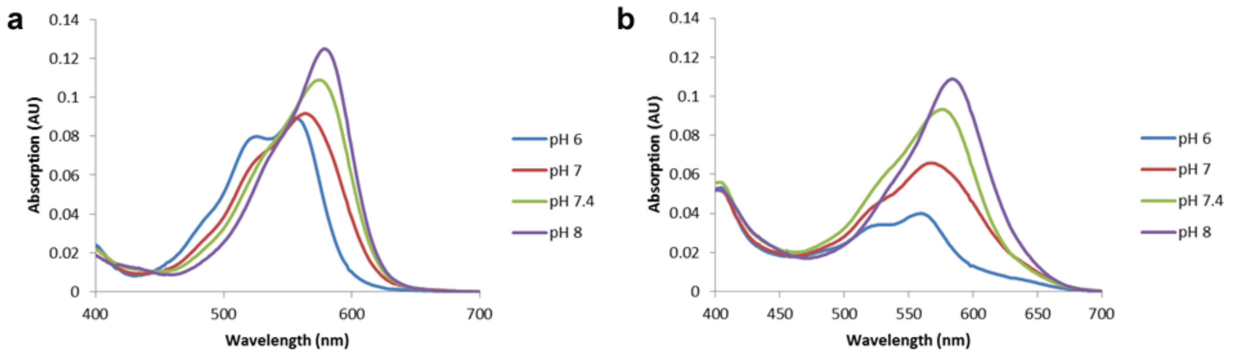

Supplementary Figure 6: (a) pH-dependent optical absorption spectrum of SNARF-5F solution. (b) pH-dependent optical absorption spectra of SNARF-5F solution mixed with human serum albumin (4mg ml^{-1}).

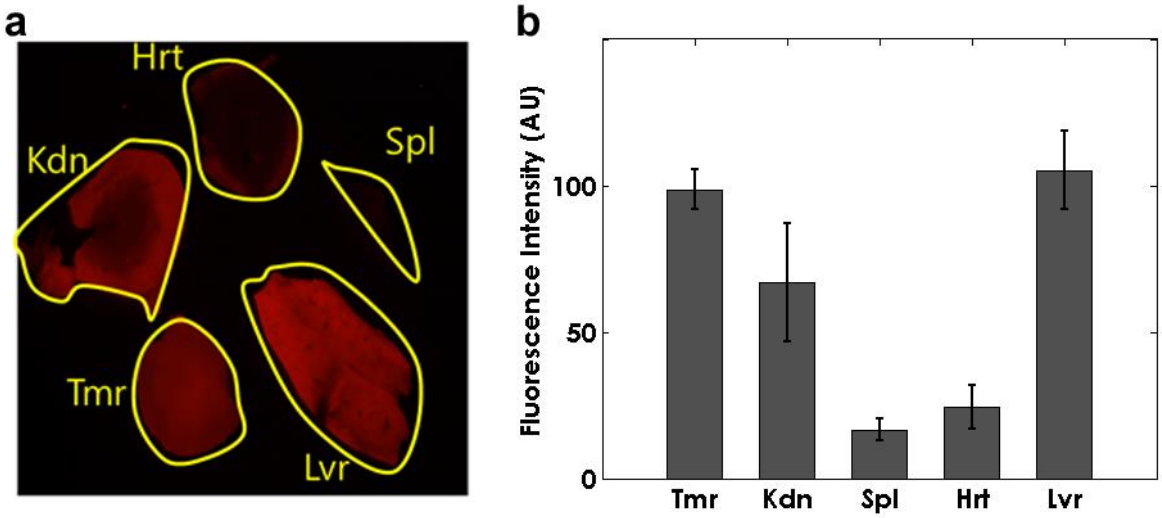

Supplementary Figure 7: Organ accumulations of SNARF-PAA NPs. (a) Fluorescence image (ex 560 nm/em 630 nm) showing SNARF-PAA NPs accumulated in the major organs and the tumor dissected from a mouse. (b) Quantified fluorescence intensities in different tissues. Error bars represent standard deviations (*n*=3). Tmr: tumor, Kdn: kidney, Spl: spleen, Hrt: heart, and Lvr: liver.

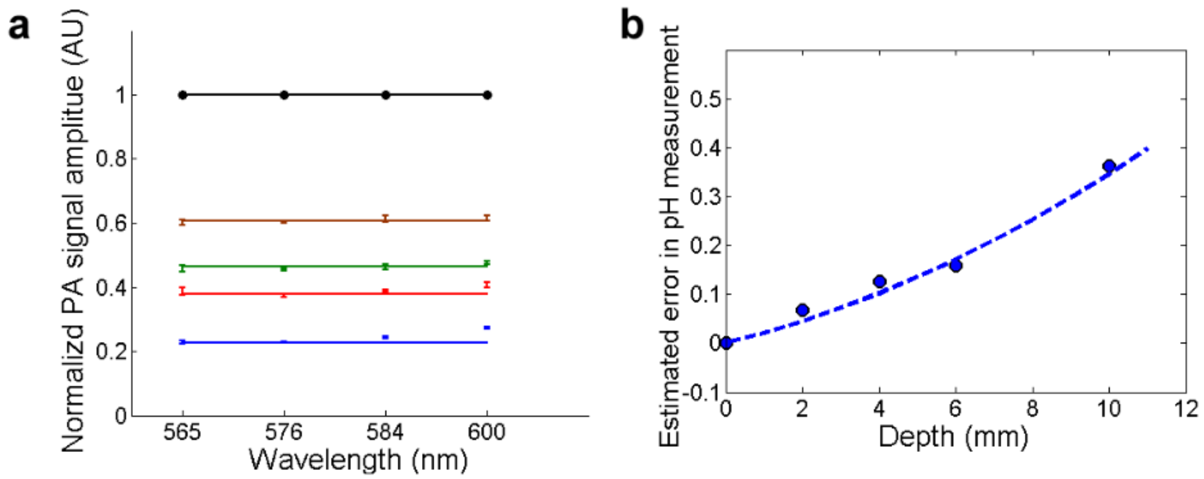

Supplementary Figure 8: Error in pH quantification due to the spectroscopic difference in optical attenuation in tissue. (a) At different depths (2, 4, 6, and 10 mm), the PA signal amplitudes at the four wavelengths for ratiometric PAI are normalized to the measurements at depth=0 (when the object was not covered by any tissue). The spectroscopic differences in the normalized PA signal amplitudes become larger when the image depth increases. Error bars represent standard deviations (*n*=3). (b) The estimated error in pH quantified by ratiometric PAI as a function of the imaging depth. This error in pH measurement as a result of the wavelength-dependent optical attenuation increases with the imaging depth. The dash line shows the result of Gaussian fitting.

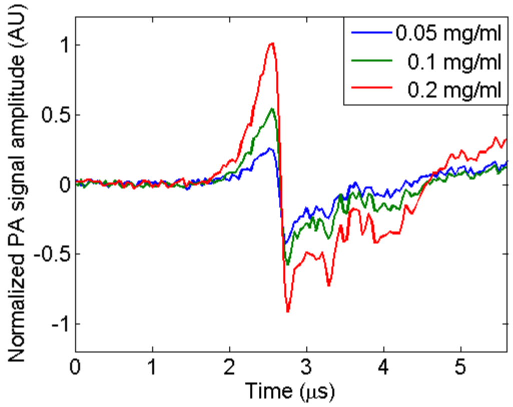

Supplementary Figure 9: PA signals of SNARF-PAA NP solutions embedded 6-mm deep in optically scattering tissue. The signals correspondent to different NP concentrations (0.2 mg ml^{-1}, 0.1 mg ml^{-1}, and 0.05 mg ml^{-1}) can all be detected with good signal-to-noise ratio.

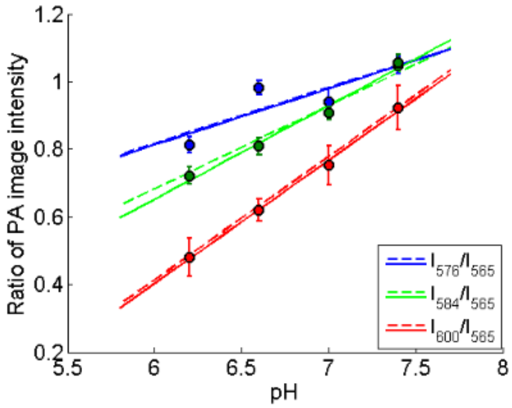

Supplementary Figure 10: Quad-wavelength ratiometric calibration lines measured by the PAI system built on the Verasonics platform. The solid lines (i.e. 576 nm/565 nm, 584 nm/565 nm, and 600 nm/565 nm) are the linear fittings of the measurements at four different pH levels (i.e. pH 6.2, pH 6.6, pH 7, and pH 7.4). The dashed lines are the results from the V312 transducer (**Fig. 4a**) for comparison. Error bars represent standard deviations (n=50).

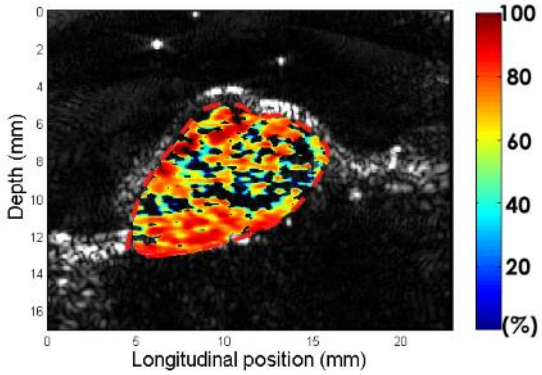

Supplementary Figure 11: PA hemoglobin oxygen saturation (sO_2) image in the tumor *in vivo* before the injection of SNARF-PAA NPs.

Supplementary References

1. Buck SM, Koo YE, Park E, Xu H, Philbert MA, Brasuel MA, *et al.* Optochemical nanosensor PEBBLEs: photonic explorers for bioanalysis with biologically localized embedding. *Curr Opin Chem Biol* 2004, 8(5): 540-546.
